# Casual blood pressure of adolescents attending public secondary schools in Maiduguri, Nigeria

**DOI:** 10.1186/s40885-015-0026-5

**Published:** 2015-09-03

**Authors:** Adetoyeje Y. Oyeyemi, Muhammad A. Usman, Adewale L. Oyeyemi, Olabode A. Jaiyeola

**Affiliations:** Department of Physiotherapy, College of Medical Sciences, University of Maiduguri, P.M.B 1069, Maiduguri, Nigeria; Department of Physiotherapy, University of Maiduguri Teaching Hospital, Maiduguri, Nigeria

**Keywords:** Undiagnosed hypertension, Cardiovascular parameters, Adolescents

## Abstract

**Introduction:**

Although evidence exists that adolescents’ hypertension could lead to adults’ hypertension, it is a general belief that measures for early detection and treatment of this condition is seldom undertaken especially in medically underserved communities such as Maiduguri, Northeastern Nigeria. This study investigated the prevalence of undiagnosed hypertension among adolescents in Maiduguri, Nigeria, and explored the association between undiagnosed hypertension and adolescents’ physical characteristics and parental socio-demographic factors.

**Methods:**

Participants’ physical characteristics were assessed, and information on their socio-demographics including parental socioeconomic status were obtained. Blood pressures and heart rates of the participants were also measured three times at 5–10-min period.

**Results:**

The prevalence of undiagnosed hypertension among the students was 13.2 %, and overwhelming majority (82.4 %) of the hypertensive students were in the prehypertensive stage, while 17.6 % were in the stage 1 classification of hypertension. Higher prevalence of undiagnosed hypertension was observed for the females compared to the male adolescents (*X*^2^ = 15.49, *p* = 0.001), and presence of undiagnosed hypertension is positively but tenuously linked to age (*r* = 0.11, *p* = 0.01), body mass index (*r* = 0.10, *p* = 0.01), and parental income (*r* = 0.26, *p* = 0.02) of the students.

**Discussion:**

This study suggests that any effective programs designed to mitigate undiagnosed hypertension among adolescents in this city should include strategies that address possible barriers to physical activity and exercise among female adolescents in the society.

## Introduction

In the past four decades, increased urbanization and changing lifestyles in Africa have raised the incidence of non-communicable chronic diseases (NCDs) to near epidemic proportions [1]. These diseases accounted for 22 % of the total deaths in African region in the year 2000, and cardiovascular diseases alone accounted for 9.2 % of the total deaths, killing even more than malaria [2]. It has been projected that up to three quarters of the world’s hypertensive population will be in economically developing countries by the year 2025 [2].

Hypertension is the most common cardiovascular disease in the whole world [3]. This condition has been identified as a major risk factor for stroke, congestive heart failure, renal disease, and myocardial infarction. In some developed countries, the prevalence rate of undiagnosed hypertension among adolescents was 37.7, 38.4, 41.7, 48.7, 55.3, 44, and 28 % in Italy, Sweden, England, Finland, Germany, the entire Europe and the United States, respectively [3], despite the modern and accessible health infrastructures in these developed world. Almost 90 % of the adolescents with undiagnosed hypertension in these countries have essential hypertension [4], and this is becoming increasingly common along with overweight, insulin resistance, and dyslipidemia [5] while about 0.1 % of hypertension in adolescents is due to secondary causes mostly renal disease [6].

In a study on Nigerian children, both diastolic and systolic pressures were consistently high in a substantial number of the Nigerian children [7] and was attributed more to the environment than genetic factors although family history might play some role. Insufficient breastfeeding, overweight or obesity, smoking, alcohol abuse, lack of physical fitness, and socioeconomic status have been associated with hypertension and so are diets high in sodium, potassium, calcium, magnesium, and diabetes mellitus [8, 9].Whereas another study shows low prevalence from 0–10.5 % of adolescents between the age of 12 to 18, and the higher the age the higher the prevalence [10].

Although evidence exist that adolescents’ hypertension can lead to adult hypertension [11], adolescents who have high blood pressure are at risk of developing cardiovascular complications and organ damage when other risk factors are also present [12]. Despite this, measurement of blood pressure in children is usually delayed or even seldom performed especially in medically underserved regions such as Northeastern Nigeria thus preventing the early detection of the problem and its subsequent treatment [13]. It is the consensus that early identification of risk factors, mainly among the young, may help in the primary prevention of a series of cardiovascular, neurological, and renal complications [14, 15].

However, compared to other environments where several studies on prevalence of undiagnosed hypertension among different demographic populations exist [3, 5, 16], there is still paucity of data on undiagnosed hypertension among populace of Northeastern Nigeria, with an estimated adolescents population of about 1 million [17]. The objectives of this study were therefore to determine the prevalence of undiagnosed hypertension among adolescents between 13 and 18 years of age and to explore any link between undiagnosed hypertension and the physical characteristics and parental socio-demographic status of the adolescents.

## Methods

### Participants and sampling

Participants in this study were boys and girls within the ages of 13 and 18 years who reported they have not been diagnosed with hypertension by their physician or any other health worker and who attend government-owned secondary schools in Maiduguri Metropolitan Council (MMC). This descriptive cross-sectional design study utilized a multistage sampling technique, in which the entire 14 eligible public secondary schools in Maiduguri Metropolitan Council were categorized into three groups i.e., male only, female only, and mixed schools groups. Of the total 16 schools in the city, two schools—the vocational training school and the school for the blind, were excluded from the study because they were non-conventional as others.

In the first stage, two out of the available four male only schools, two out of the available four female only schools, and three out of the six mixed schools were randomly selected. In stage 2, three arms were randomly selected out of the available five arms in the second, third, fourth, fifth, and sixth levels (year) of secondary education in the selected schools. Most students in the first level of their secondary education did not meet the age requirement for participation, and this level was therefore automatically excluded from selection at this stage. In stage 3, participants in all classes of the selected arms in the schools were invited to participate in the study if they are within the age bracket of 13 to 18 years. The sample size for this study was determined as 384 using the formula by Naing [18].

### Instruments

The instruments used for the study was a two-part data collection form, the first part of which consists of socio-demographic section that elicits information such as gender, age, marital status, ethnicity, religion, parent’s occupation, educational level, and previous diagnosis of hypertension. In the second part, measurements such as height, weight, blood pressure, and pulse rate were recorded on the form. Blood pressure was measured using an electronic sphygmomanometer (Accoson mercurial sphygmomanometer (A.C.Cossor and Son (Surgical) Ltd). Height was measured using a tape rule, and weight was measured using a portable bathroom weighing scale calibrated in kilograms (kg) from 0 to 200 kg.

### Procedure

Ethical approval of the University of Maiduguri Teaching Hospital was sought and obtained. On the day of first contact, an introductory letter that explained the research and its purpose was presented to the participants and the school principals before the commencement of the study. Prospective participants were also advised to inform and seek permission of their parents and were assured that any students whose parents decline will not be enrolled. None of the students reported any declination from their parents.

On the day of the second contact, participants sat for a 15-min period after which the blood pressure of each participant’s measurement was taken on three distinct occasions within a 15-min period and was recorded. The second and third measurements were taken for all the participants but recorded only for those found to be hypertensive at the first measurement, based on a previous study [15]. A time interval of at least 5 min each was observed before taking the second and third measurements [7]. Those who were either prehypertensive (blood pressure (BP) = 120–125/80–85) or at stage 1 hypertension (BP = >125–130/>85–90) were identified as those with undiagnosed hypertension [19].

### Data analysis

Descriptive statistics of frequencies was used to determine the prevalence of undiagnosed hypertension while mean and standard deviation was used to describe the physical characteristics such as age, BMI, and cardiovascular parameters including blood pressure and pulse rate of the participants. Spearman’s correlation coefficient was used to explore any relationship between undiagnosed hypertension and adolescents’ physical characteristics. Chi-square statistic was utilized to identify any trend in undiagnosed hypertension among the adolescents by parental socioeconomic factors.

## Results

### Socio-demographic characteristics of participants

A total of 1048 public secondary school students within Maiduguri, Borno State, participated in this study. The participants comprised 653 (62.3 %) males and 395 (37.7 %) females with a mean age and body mass index of 19.3 ± 2.4 years and 20.0 ± 3.2 kg/m^2^, respectively. About 85 % (*n* = 887, 84.6 %) of the participants were Muslims while 161 (15.4 %) were Christians. About 36.1 %, 61.0 %, and 3.0 % of the participants described their parents’ occupation as white collar, blue collar, and unemployed, respectively.

The prevalence of undiagnosed hypertension among the students was 13.2 %, and overwhelming majority (82.4 %) of the students with undiagnosed hypertension were in the prehypertension stage, and only about 17.6 % were in the stage 1 classification of hypertension. Detailed socio-demographic characteristics and prevalence of undiagnosed hypertension of the participants are shown in Table 1.

**Table 1:** Participants’ socio-demographic characteristics and prevalence of undiagnosed hypertension (*n* = 1048)

Characteristics	Values
Age	
Mean (SD)	15.6(1.6)
Range	13–18
BMI	
Mean (SD)	19.5(2.8)
Range	13.5–30.5
Body weight status (*n*, %)	
Underweight	387(36.9)
Ideal weight	621(59.3)
Overweight	35(3.3)
Obese	5(0.5)
Gender (*n*, %)	
Male	653(62.3)
Female	395(37.7)
Religion (*n*, %)	
Islam	887(84.6)
Christianity	161(15.4)
Occupation of parents (*n*, %)	
White collar	378(36.1)
Blue collar	639(61)
Unemployed	31(3)
Education of parents (*n*, %)	
Higher education	606(57.8)
Secondary education	327(31.2)
Primary education	26(2.5)
Islamic education	89(8.5)
Undiagnosed hypertension (*n*, %)	
Yes	138(13.2)
No	910(86.8)
Stages of hypertension (*n*, %)	
Prehypertension	117(82.4)
Stage 1 hypertension	25(17.6)

Table 2 shows participants’ SBP according to age percentiles while Table 3 shows participants’ DSP according to age percentile. Both the systolic and diastolic blood pressure levels according to age percentiles as indicated in the two tables show downward trend in the readings of the systolic and diastolic blood pressure. First BP measurement is higher than the second and the third measurements. The second measurement is also higher than the third measurement.

**Table 2:** Systolic blood pressure levels according to age percentiles

	Percentiles														
Age of participants	25			50			75			90			95		
	1st	2nd	3rd	1st	2nd	3rd	1st	2nd	3rd	1st	2nd	3rd	1st	2nd	3rd
13	130.00	119.00	114.00	132.50	136.00	120.00	134.50	141.00	121.25	150.00	143.00	127.00	150.00	143.00	127.00
14	133.25	119.75	117.50	138.00	128.50	128.50	147.00	141.00	136.00	148.80	141.00	141.40	–	–	–
15	120.00	112.00	119.00	132.50	128.00	126.50	133.00	129.25	141.00	163.00	133.50	140.00	–	–	–
16	119.50	121.50	112.50	129.00	126.00	120.00	127.50	134.00	137.00	144.40	137.00	145.00	181.20	151.90	148.00
17	127.00	123.00	116.00	136.00	128.00	121.50	129.00	133.00	139.50	150.00	153.00	130.00	159.70	156.00	142.60
18	127.00	125.00	109.00	130.00	132.00	125.00	143.00	138.00	127.00	147.00	143.60	136.00	153.80	159.40	139.90

**Table 3:** Diastolic blood pressure levels according to age percentiles

	Percentile														
Age of participants	25			50			75			90			95		
	1st	2nd	3rd	1st	2nd	3rd	1st	2nd	3rd	1st	2nd	3rd	1st	2nd	3rd
13	79.00	73.00	73.00	86.00	84.00	73.00	91.00	90.00	77.50	92.00	93.00	82.50	92.50	93.00	83.50
14	81.50	84.75	75.25	90.00	88.50	82.50	93.00	90.00	86.00	96.00	91.80	86.90	–	–	–
15	88.75	83.75	82.00	91.50	89.00	83.50	96.75	95.00	92.00	101.60	104.80	92.20	–	–	–
16	82.00	80.00	72.00	88.00	84.00	79.00	98.50	86.00	84.00	105.00	96.00	90.00	108.00	103.90	101.20
17	83.25	75.00	76.00	84.00	82.00	80.00	98.00	84.50	82.00	99.00	88.90	87.80	99.00	97.75	90.85
18	81.00	77.00	73.00	86.00	85.00	77.00	90.00	91.00	86.50	99.80	98.00	95.60	109.40	101.00	96.00

### Trends and relationships in prevalence

A significantly higher prevalence of undiagnosed hypertension was observed for the female compared to the male adolescents (*X*^2^ = 15.49, *p* = 0.001). No statistically significant trend was found in the prevalence of undiagnosed hypertension by tribe, religion, parental occupation, and educational level (Table 4). For all the participants, the prevalence of undiagnosed hypertension was significantly albeit poorly correlated with age (*r* = 0.11, *p* = 0.01), body mass index (*r* = 0.10, *p* = 0.01), and parents’ income (*r* = 0.26, *p* = 0.02).

**Table 4:** Chi-square statistics for prevalence undiagnosed hypertension and socio-demographic variables

Characteristics	Prevalence of undiagnosed hypertension			
	Yes (%)	No (%)	*X* ^2^	*p* value
Sex			15.49	0.001
Male	41.4	63.6		
Female	58.6	36.4		
Tribe			2.05	0.727
Hausa\Fulani	44.8	47.0		
Kanuri\Shuwa	35.6	30.6		
Others	19.5	22.4		
Religion			2.64	0.104
Islam	79.3	86.0		
Christianity	20.7	22.4		
Occupation of parents			3.54	0.1
White collar	42.5	35.8		
Blue collar	57.5	61.4		
Unemployed	0.0	2.8		
Education of parents			3.94	0.266
Higher education	64.4	56.2		
Secondary education	21.8	32.1		
Primary education	3.4	2.2		
Islamic education	10.3	9.5		

## Discussion

The main objective of this study was to investigate the prevalence and correlates of undiagnosed hypertension and to determine the physical characteristics and parental socio-demographic factors that could influence undiagnosed hypertension among adolescents in Maiduguri, Borno State. This study revealed a 13.2 % prevalence of undiagnosed hypertension among the adolescents. This prevalence rate is higher than the rates of 5.6 %, 6.3 %, 9.4 %, and 9.6 % reported in similar studies carried out in Iran [7], China [20], Brazil [21], and Tunisia [22], respectively. The prevalence rate in the present study is however lower than the rates reported in affluent societies including Italy (37.7 %), Sweden (38.4 %), England (41.7 %), Finland (48.7 %), and Germany (55.3 %) [3].

Our finding shows undiagnosed hypertension tend to occur more frequently in the females (58.6 %) than in the male students (41.4 %), and this finding is comparable to finding in a study of Tunisian adolescents [23] that shows higher prevalence in adolescent girls than in boys (51 % vs. 49 %). This finding is not in consonance with several studies on Nigerian population with age of 15 and above which shows higher prevalence [11, 24–26] or no difference in prevalence by gender [27].

Based on a priori comparison with previous studies of adolescents in Nigeria [11, 28], the reason for the relatively higher prevalence for undiagnosed hypertension found in the present is unclear. Maiduguri city is regarded as the heart of insurgency activities which started 4 years ago in Nigeria. It can be argued that the higher prevalence rate observed in the present study may be attributed to the prevailing anxiety and fear among the general populace including the children and youths in the city, as a result of the security situation at the time of this study.

The reason for the higher prevalence of undiagnosed hypertension in females than in males is also unclear unlike a study which shows higher prevalence in systolic but not diastolic prehypertension in males than females among Nigerian adolescents [11]. Physical activity has been documented to confer protection against the development and onset of hypertension [29, 30], and adolescent females in this society tend to be less encouraged to play or engage in moderate to highly intense physical activity especially outdoor when compared to their male counterparts. In this society, females may join their mothers in light household chores whose activity intensity and rigor may not equate those possible during outdoor plays.

The differential prevalence rate by gender may be an indication that sex based differences in physical activity behavior may have started to show at adolescents’ stage. Higher prevalence among the females than males suggests the need to investigate possible differences in physical activity behavior between the sexes that might already manifest in adolescence. This study shows that strategies to address early detection and remediation of hypertension in adolescents must include gender-specific intervention.

A priori, we compared our results with those of Balogun et al. who reported on the casual blood pressure of elementary school student in South Western part of Nigeria over two decades ago [30, 31]. In the study, body weight was a strong determinant of blood pressure, while parental socioeconomic status did not influence the blood pressure of elementary and secondary school students. Our study however shows a tenuous link between undiagnosed hypertension and BMI and parental socioeconomic status. Nevertheless, the present study affirms the need for early detection and intervention for adolescents with cardiovascular risk factors.

### Study limitations

One obvious limitation of this study though is the inability to control for other factors that could affect blood pressure such as engaging in physical activity within 2 h prior to the test, temperature, diet, emotional state, physical state, and medications [32]. Conclusions made from direct comparison of the prevalence rate as found in the present study, and the rate as reported in other studies made above should also be drawn with caution. This is because in Balogun et al.’s study [31], high casual blood pressure was based on 95th percentile cutoff, while in the present study, prevalence rate was based on the blood pressure categorizations as defined by the National High Blood Pressure Education Program (NHBPEP) [19].

## Conclusion

Our study results shows a fairly high prevalence rate of undiagnosed hypertension among adolescents in public secondary schools in Maiduguri and a slightly higher prevalence among females than males. The findings that show a substantial number of adolescents in public secondary schools in this city are at risk for developing hypertension at later years suggest the need for extensive campaign and intervention through education on diet including salt and fat intake and consumption of processed food and physical activity at this adolescent stage or earlier. Any such intervention through physical activity should include strategies that address possible barriers to physical activity and exercise restrictions of female adolescents in the society.

## Abbreviations

BMI: body mass index
BP: blood pressure
MMC: Maiduguri Metropolitan Council
SD: standard deviation
